# Ossification variants of the distal femoral condyle: longitudinal 3 T MRI evidence of progression to juvenile osteochondritis dissecans in asymptomatic siblings of patients with JOCD

**DOI:** 10.1007/s00256-026-05254-8

**Published:** 2026-06-08

**Authors:** Abdul Wahed Kajabi, Saumith Bachigari, Theo Nguyen, Eisa Hedayati, Karsten Knutsen, Aileen Ugurbil, Anna Menezes, Michael Newcome, Rohan Raikar, Marc A. Tompkins, Takashi Takahashi, Bradley J. Nelson, Kevin G. Shea, Jutta M. Ellermann

**Affiliations:** 1 Center for Magnetic Resonance Research, University of Minnesota, Minneapolis, MN USA; 2 Department of Radiology, University of Minnesota, Minneapolis, MN USA; 3 Laboratory of Single Cell Genomics and Population Dynamics, The Rockefeller University, New York, NY USA; 4 The Tri-Institutional MD-PhD Program, New York, NY USA; 5 Department of Orthopedic Surgery, University of Minnesota, Minneapolis, MN USA; 6 Stanford Children’s Hospital, Palo Alto, CA USA

**Keywords:** Pediatric MRI, Osteochondrosis, Osteochondritis dissecans, Ossification variants, OV, Juvenile osteochondritis dissecans, JOCD, Endochondral ossification, Skeletal development

## Abstract

**Objective:**

Ossification variants (OVs) of the femoral condyles are traditionally regarded as benign developmental findings distinct from juvenile osteochondritis dissecans (JOCD). We aimed to characterize the longitudinal MRI behavior of OVs and JOCD lesions in asymptomatic siblings of JOCD patients.

**Materials and methods:**

In this HIPAA-compliant longitudinal pilot study, seven asymptomatic siblings of JOCD patients underwent serial 3 T bilateral knee MRI. Two fellowship-trained musculoskeletal radiologists independently assessed 56 studies for bone marrow edema, lesion location, and MRI-defined category (OV or JOCD).

**Results:**

OV and MRI-defined JOCD lesions were identified in 21 of 28 condyles (75%, 95% CI: 56.6–87.3%), while 7 condyles (25%, 95% CI: 12.7–43.4%) remained normal throughout follow-up. Six condyles demonstrated MRI-defined JOCD lesions at one or more timepoints. Three OV lesions evolved over time: two progressed to MRI-defined JOCD but remained clinically silent, and one progressed from OV to MRI-defined JOCD and subsequently to clinically manifest JOCD requiring surgery. Using Generalized Linear Mixed model, a statistically significant association was found between bone marrow edema and MRI-defined category (F = 31.73, p < 0.001). OV lesions showed absent or trace edema, whereas JOCD showed definite edema. Inter-reader agreement using Cohen’s Kappa was moderate to substantial between the radiologists (κ = 0.479–0.739, 95% CI: 0.314–0.633, 0.644–0.845, p < 0.001).

**Conclusions:**

In siblings of patients with JOCD, OV lesions are common and may represent dynamic MRI phenotypes along a continuum of epiphyseal ossification abnormalities, with occasional progression to MRI-defined JOCD and rare progression to clinically manifest JOCD.

## Introduction

Distinguishing ossification variants (OVs) from early juvenile osteochondritis dissecans (JOCD) lesions on pediatric knee MRI is increasingly important as MRI is more frequently performed in children for persistent knee pain previously attributed to “growing pains”, sports-related overuse or injury, and other indications that yield incidental epiphyseal findings. Radiologists are often required to determine whether an abnormality of the distal femoral ossification front on MRI represents a benign developmental OV, warranting reassurance or an early JOCD lesion that may prompt imaging follow-up, and orthopedic referral [1–7]. This distinction is particularly critical in higher-risk populations, including children with a familial association of JOCD [8, 9], such as siblings of affected patients, as well as youth participating in high-impact sports [10, 11], in whom epiphyseal abnormalities on MRI may represent ossification variants or silent JOCD lesions before symptom onset.

Clinically manifest JOCD is a pathologic growth disturbance of the secondary physis during skeletal development [12–14] typically presenting with knee pain and/or mechanical symptoms. It predominantly affects adolescent boys and most commonly but not exclusively involves the central aspect of the medial femoral condyle [12, 15, 16]. Across disease stages, JOCD lesions share two key MRI features: an irregular or delayed ossification front and adjacent subchondral bone marrow edema, reflecting disturbance of normal endochondral ossification. In contrast, OV lesions of the femoral condyles are described as benign developmental variants of ossification [4–6, 17]. They are described to typically occur more posteriorly at the medial or lateral femoral condyle, may mimic early JOCD lesions, but classically remain asymptomatic and lack bone marrow edema beyond expected red marrow [5, 6]. OV lesions are common, with reported prevalences of 12.6–22% in pediatric populations [3], and therefore generally regarded as normal variants rather than precursors of disease [3–6]. However, the relationship between OV and JOCD remains incompletely understood. Laor et al. and others have emphasized the need for longitudinal imaging to determine whether some OV lesions may evolve toward JOCD [5]. Conversely, early JOCD lesions may normalize with skeletal maturation, underscoring that classification at a single time point may be misleading.

Siblings of patients with JOCD represent a particularly informative cohort because they share genetic and environmental risk factors [12]. In this study, the siblings were clinically asymptomatic and imaged at an earlier developmental stage than the affected child, providing a window into the earliest manifestations of epiphyseal abnormalities. To date, the longitudinal behavior of OVs and early JOCD lesions on MRI in siblings has not been systematically characterized.

Accordingly, the purpose of this longitudinal 3 T MRI pilot study, was to follow asymptomatic siblings of patients with JOCD over multiple timepoints to: (i) characterize abnormalities of the distal femoral ossification front and bone marrow, with emphasis on differentiating OV from JOCD lesions; (ii) assess inter-reader agreement for these imaging findings; and (iii) determine the frequency with which lesions initially classified as OV, resolve, remain stable, or progress to MRI-defined JOCD or clinically manifest JOCD. By focusing on this familial JOCD cohort, we aim to evaluate whether there is a spectrum of epiphyseal ossification abnormalities, including OV and JOCD lesions, that may underlie clinically manifest JOCD pathogenesis and may inform risk stratification, imaging follow-up, and timely orthopedic referral. The current study represents a small pilot cohort of 7 siblings; therefore, precise frequency estimates carry wide uncertainty and should be interpreted with caution.

## Materials and methods

### Study participants

This prospective longitudinal pilot study was HIPAA-compliant, approved by the IRB, and conducted with written informed consent obtained from all study participants and their legal guardians. All included participants were initially asymptomatic siblings of patients diagnosed with clinical JOCD at our institution. Under the IRB-approved protocol, siblings were recruited by the pediatric orthopedic surgeon treating the index JOCD patient. The siblings underwent imaging at our institution between May 2018 to December 2023. MRI data were collected serially (median 8 months, interquartile range = 14 months), with each sibling undergoing between two to five follow-up scans (Table 1). Both femoral condyles were assessed bilaterally as part of the IRB-approved research protocol; while JOCD predominantly affects the medial femoral condyle [18], the lateral femoral condyle represents the second most common location[19] and was assessed accordingly.

**Table 1:** Demographics of the study participants

Participant	Gender	Timeline	Symptoms	Age (years)	Bone marrow edema				MRI-defined category			
					Left knee		Right knee		Left knee		Right knee	
					LFC	MFC	LFC	MFC	LFC	MFC	LFC	MFC
Sibling 1	Male	Baseline	None	9.5	Trace	Trace	Trace	Trace	OV	OV	OV	OV
		FUP 1	None	10.1	Trace	Trace	Trace	Trace	OV	OV	OV	OV
		FUP 2	None	10.5	Trace	Trace	Edema	Edema	OV	OV	OV	OV
		FUP 3	None	11.0	Normal	Edema	Trace	Edema	OV	JOCD	OV	JOCD
Sibling 2	Male	Baseline	None	6.4	Normal	Trace	Trace	Normal	OV	OV	OV	OV
		FUP 1	None	7.0	Trace	Trace	Trace	Trace	OV	OV	OV	OV
		FUP 2	None	7.4	Trace	Trace	Trace	Trace	OV	OV	OV	OV
		FUP 3	None	8.0	Trace	Trace	Trace	Trace	OV	OV	OV	OV
		FUP4	None	10.2	Trace	Trace	Normal	Trace	OV	OV	OV	OV
Sibling 3	Female	Baseline	None	9.7	Normal	Normal	Normal	Normal	Normal	Normal	Normal	Normal
		FUP1	None	10.4	Normal	Normal	Normal	Normal	Normal	Normal	Normal	Normal
Sibling 4	Female	Baseline	None	10.2	Normal	Edema	Normal	Edema	Normal	JOCD	Normal	JOCD
		FUP 1	None	11.1	Normal	Normal	Normal	Normal	Normal	Normal	Normal	Normal
		FUP 2	None	11.2	Normal	Trace	Normal	Trace	Normal	OV	Normal	OV
		FUP 3	None	12.0	Normal	Normal	Normal	Normal	Normal	Normal	Normal	OV
		FUP4	None	14.4	Normal	Normal	Normal	Normal	Normal	Normal	Normal	Normal
Sibling 5	Male	Baseline	None	8.2	Trace	Trace	Trace	Trace	OV	OV	OV	OV
		FUP 1	None	9.0	Edema	Trace	Trace	Trace	JOCD	OV	OV	OV
		FUP 2	None	9.2	Edema	Trace	Trace	Trace	JOCD	OV	OV	OV
		FUP 3	None	9.8	Edema	Trace	Trace	Trace	JOCD	OV	OV	OV
		FUP4	Yes	11.9	Edema	Trace	Edema	Trace	JOCD	OV	OV	OV
Sibling 6	Male	Baseline	None	10.6	Trace	Trace	Trace	Trace	OV	OV	OV	OV
		FUP 1	None	11.3	Normal	Trace	Normal	Trace	OV	OV	OV	OV
		FUP 2	None	12.1	Normal	Trace	Normal	Normal	Normal	OV	Normal	Normal
		FUP 3	None	14.6	Normal	Normal	Normal	Normal	Normal	Normal	Normal	Normal
Sibling 7	Female	Baseline	None	6.5	Normal	Trace	Normal	Trace	Normal	OV	Normal	OV
		FUP 1	None	7.8	Normal	Normal	Edema	Trace	Normal	Normal	JOCD	OV
		FUP 2	None	10.3	Normal	Normal	Normal	Normal	Normal	Normal	Normal	Normal

*FUP* follow-up, *LFC* lateral femoral condyle, *MFC* medial femoral condyle, *OV* ossification variant, *JOCD* juvenile osteochondritis dissecans

The inclusion criteria required that participants, i) had no history of clinical symptoms (e.g., pain, clicking, or locking), ii) were siblings of patients diagnosed with clinically manifest JOCD, and iii) were less than 18 years old.

### MRI protocol

Imaging was performed on a whole-body 3 T MRI system (Prisma Fit, Siemens Healthineers) using a dedicated knee coil (birdcage single-transmit with a 15-channel receive array, Quality Electrodynamics). The MRI protocol included standard clinical turbo spin echo (TSE) sequences: axial T1- and fat-suppressed T2-weighted, sagittal proton density (PD)-weighted with and without fat-suppression, and coronal T1- and fat-suppressed T2-weighted scans. All the sequences and their corresponding parameters are listed in Table 2.

**Table 2:** MRI acquisition parameters

Acquisition parameter	Axial		Sagittal		Coronal	
	T1w	T2w FS	PDw	PDw FS	T1w	T2w FS
Repetition time (TR)(ms)	584	3860	1810	2000	786	3860
Echo time (TE) (ms)	8.7	36	37	32	9.3	47
Flip angle (degrees)	140	150	150	150	150	150
Pixel BW (Hz/pix)	240	195	240	240	200	200
Field of view (FOV) (mm^2^)	140 × 140	130 × 130	110 × 110	110 × 110	110 × 110	110 × 110
Matrix size	384 × 288	256 × 233	256 × 218	256 × 218	320 × 224	320 × 224
Resolution^a^ (mm^2^)	0.36 × 0.36	0.51 × 0.51	0.43 × 0.43	0.43 × 0.43	0.34 × 0.34	0.34 × 0.34
Slice thickness (mm)	3.0	3.0	3.0	3.0	3.0	3.0
Number of slices	41	31	31	31	31	31
Echo train length	4	11	5	5	2	11
Number of averages	1	1	1	2	1	2
Imaging time (min:s)	01:15	01:17	01:21	02:54	01:01	02:22

*PDw* proton-density-weighted, *FS *fat-suppressed, *BW *Bandwidth

### Review of MRI studies

**Fig. 1:**
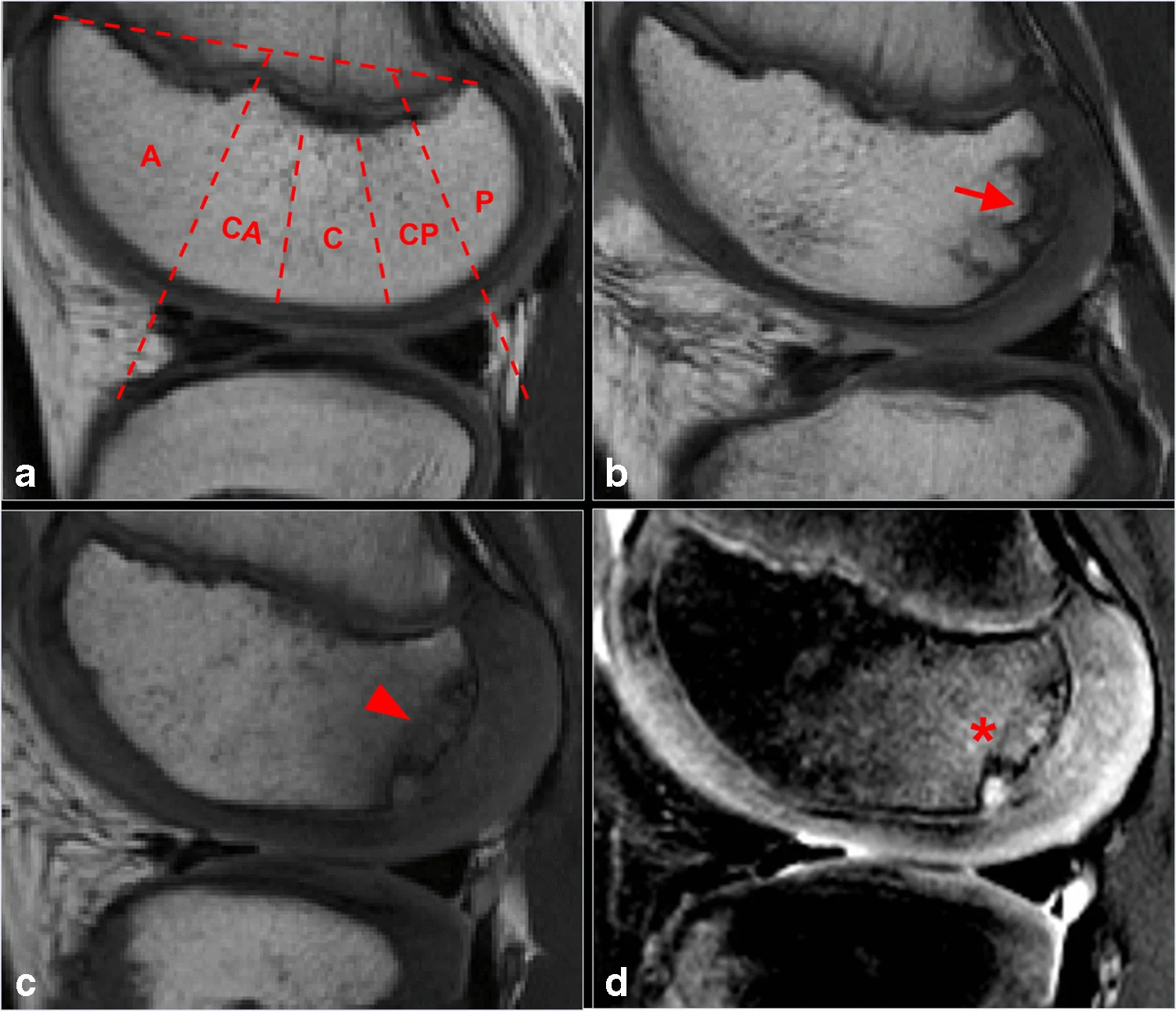
PD-weighted sagittal TSE images; **A**-**C** without and **D** with fat suppression from an a nine-year-old girl **A**, an eight-year-old boy **B**, and a ten-year-old boy **C**, **D**. **A** illustrates the predefined distal femoral condyle location map used by both radiologists, **B** an OV lesion (arrow, B), **C** a JOCD lesion (arrowhead, C), and **D** associated bone marrow edema (asterisk, D). A = anterior, CA = central-anterior, C = central, CP = central-posterior, P = posterior; the latter three used by the radiologists to describe the lesion locations. OV = ossification variant, JOCD = juvenile osteochondritis dissecans

All baseline and follow-up MRI examinations were independently reviewed by two fellowship-trained musculoskeletal radiologists (*blinded*, with 18 years of experience, and *blinded*, with 10 years of experience). Using published criteria [5, 6], images were first assessed for an abnormality of the distal femoral ossification front and, when present, characterized by (i) bone marrow edema (none, trace, or definite edema relative to normal red marrow), (ii) location of the ossification-front abnormality (central, central–posterior, or posterior) (Fig. 1A), and (iii) overall MRI-defined category (OV or JOCD) (Fig. 1). Condyles without an ossification-front abnormality were classified as normal.

Bone marrow signal was evaluated on PD- or T2-weighted fat suppressed images relative to normal red marrow. Trace edema was defined as subtle hyperintensity not exceeding red marrow, whereas definite edema was defined as hyperintensity exceeding red marrow.

Consistent with published MRI criteria [5, 6], an OV was defined as an irregularity of the distal femoral ossification front with absent or only trace bone marrow edema not exceeding red marrow. OVs are traditionally described as located more posteriorly and considered developmental variants of endochondral ossification rather than disease.

JOCD lesions were defined as delay or irregularity of the ossification front accompanied by bone marrow edema exceeding red marrow, typically in more central locations. MRI categories were treated as phenotypic imaging descriptors rather than disease labels. Clinically manifest JOCD was assigned only when symptoms developed in conjunction with a JOCD lesion on MRI. Throughout the manuscript, *the terms OV and JOCD refer exclusively to MRI-defined findings, whereas clinically manifest JOCD refers to symptomatic disease*.

### Statistical analysis

Statistical analyses were performed in SPSS (IBM Corp, Version 29.0. Armonk, NY) using Generalized Linear Mixed model to evaluate the association between edema and MRI-defined category outcome, representing imaging criteria reported by the two radiologists. The dataset comprised seven subjects, with both knees and medial and lateral femoral condyles assessed independently (28 observations in total). To account for within-subject data dependency, condyle-level identifiers were included as random effects in the model. In addition, crosstabulation tables were generated (restricted to baseline data) to provide item-wise association and visualize the distribution of responses across categories. All statistical tests were two-tailed, and significance was determined at a p-value of less than 0.05.

Inter-rater reliability between the two radiologists who independently evaluated each study was assessed using Cohen’s kappa to quantify the consistency of their measurements.

## Results

### Demographics and prevalence

Seven siblings of patients diagnosed with clinically manifest JOCD underwent bilateral knee MRI examinations, resulting in 14 knee MRI studies (28 femoral condyles) scanned between two to five times over the course of this study, with a median interval of 8 months (interquartile range = 14 months) (Table 1). The cohort included seven siblings of JOCD patients (three female, mean age at baseline = 8.7 ± 1.7 years, range = 6–10 years). All enrolled participants had no clinical symptoms at baseline, and only one developed clinically manifest JOCD with symptoms of pain and locking requiring surgical treatment during the follow-up period. Sibling 1 demonstrated bilateral LFC and MFC OV at baseline, progressing to MFC MRI-defined JOCD at follow-up; Sibling 4 demonstrated bilateral MFC MRI-defined JOCD at baseline, with subsequent resolution; sibling 7 demonstrated LFC MRI-defined JOCD on the right knee at first follow-up with complete resolution at final visit; and sibling 5 demonstrated LFC JOCD from follow-up 1 onward, with the only sibling who developed clinical symptoms (age 11.9) (Table 1). Importantly, JOCD lesions were identified in both the MFC and LFC across the cohort. Given the small size of this pilot cohort, no conclusions can be drawn regarding the relative frequency of medial versus lateral condyle involvement compared to population-based estimates in the literature, including Nguyen et al. 2025 and 2026 [19–21].

**Fig. 2:**
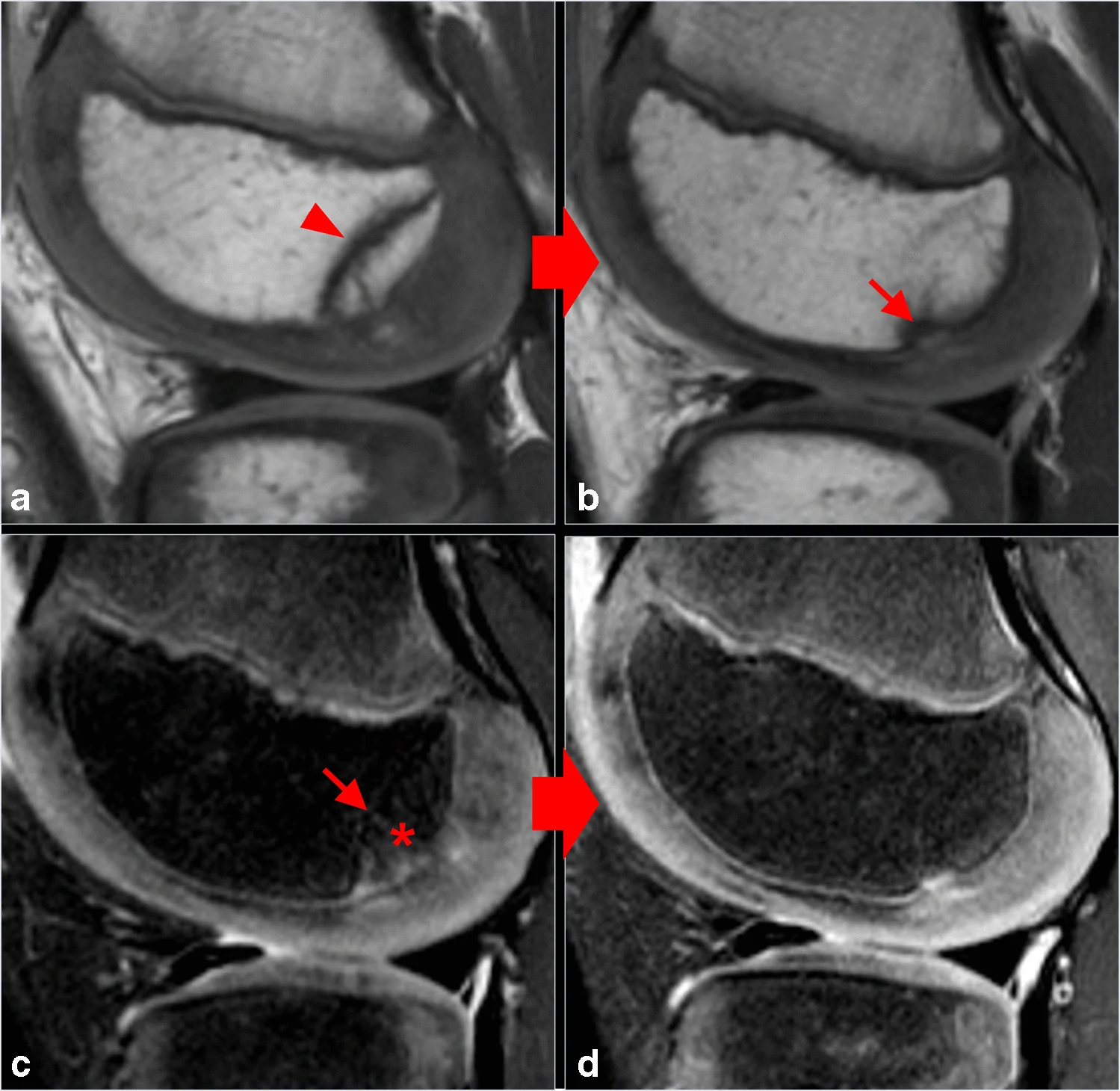
PD-weighted sagittal TSE images without **A**, **B** and with fat saturation **C**, **D** from an eight-year-old male sibling of a JOCD patient demonstrate an OV lesion (arrowhead, A) with trace edema–not exceeding signal intensity of red marrow (asterisk, C) at baseline **A**, **C** and its near resolution on follow-up **B**, **D** in the lateral femoral condyle. Large, two-piece “puzzle-piece” OV lesion (arrowhead, A) with trace marrow edema and no fluid at the interface (arrow, C), consistent with a classic OV lesion. Follow-up **B**, **D** show near complete resolution with only minimal residual contour irregularity (arrow, B) of the lateral femoral condyle. JOCD = juvenile osteochondritis dissecans, OV = ossification variant

**Fig. 3:**
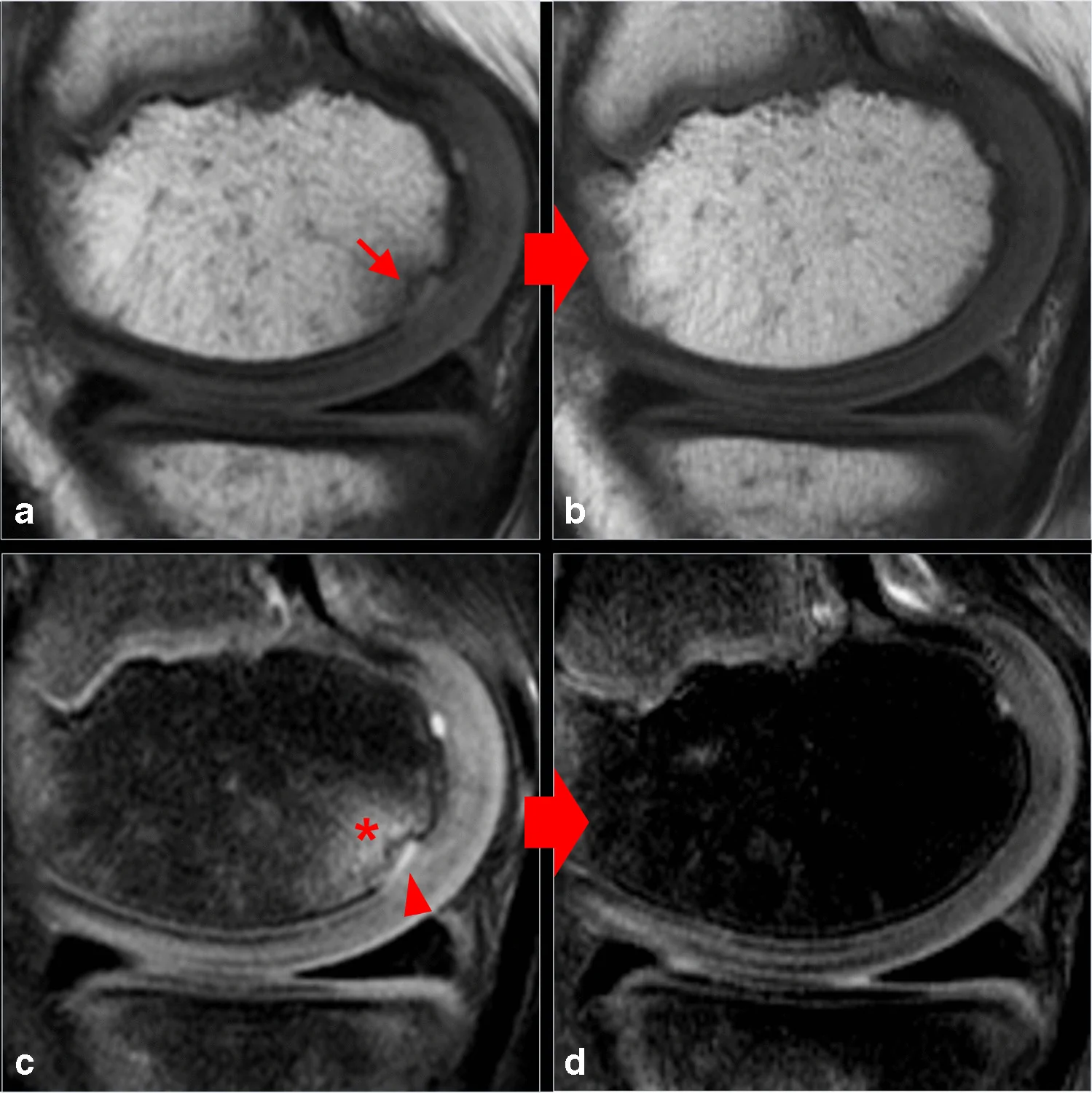
PD-weighted TSE images without **A**, **B** and with fat saturation **C**, **D** from a 10-year-old female sibling of a JOCD patient demonstrate marrow edema consistent with a JOCD lesion at baseline **A**, **C** and complete resolution on follow-up **B**, **D**. The lesion on the medial femoral condyle (arrow, A) demonstrates definite edema with signal intensity beyond red marrow (asterisk, C) deep to the concave delay of the ossification front in central-posterior location, diagnostic of a JOCD lesion. Also note disruption of the secondary physis (arrowhead, C). Complete healing with resolution of findings was observed on the follow-up scan. The sibling remained clinically asymptomatic throughout the study period. JOCD = juvenile osteochondritis dissecans

**Fig. 4:**
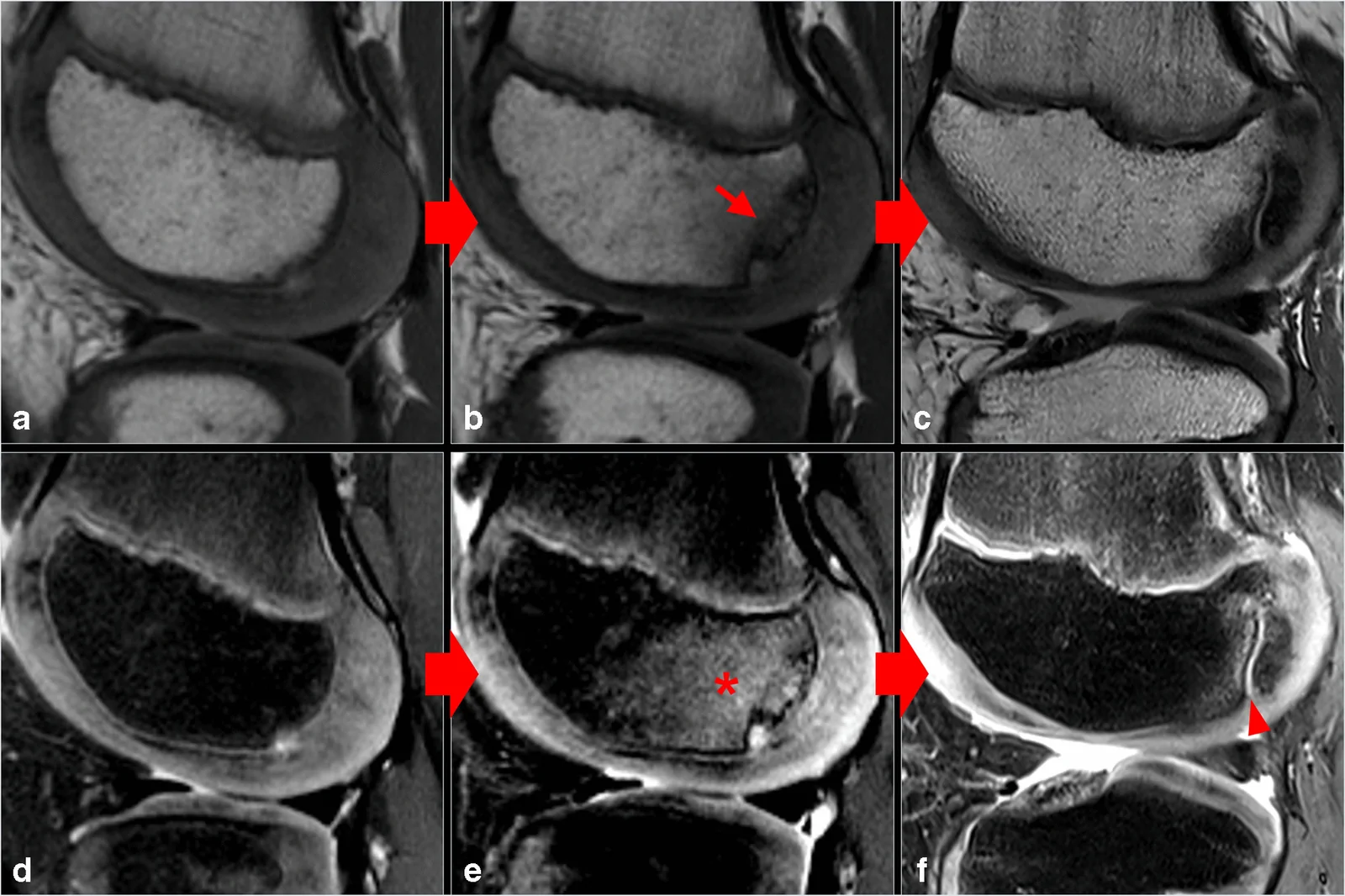
Representative PD-weighted TSE images without fat suppression **A**-**C** and with fat-suppression **D**-**F**) of an 8-year-old male sibling of a JOCD patient demonstrate progression of an OV lesion at baseline **A**, **D**) to a JOCD lesion with onset of symptoms (clinically manifest JOCD) at 12-month follow-up **B**, **E**), and subsequent development of an unstable JOCD lesion at 44-month follow-up **C**, **F**). Baseline shows a subtle lateral femoral condyle OV lesion without significant marrow edema (A, D). At 12 months, focal delay/irregularity of the ossification front (arrow, B) with marked marrow edema (asterisk, E) is present, consistent with a JOCD lesion. At 44 months, fluid at the lesion–parent bone interface (arrowhead, F) indicates instability; associated cartilage fissuring was present on an adjacent slice (not shown), and the patient required surgical treatment. OV = ossification variant, JOCD = juvenile osteochondritis dissecans

Across all condyles, OV (Fig. 2) and MRI-defined JOCD (Fig. 3) lesions were identified in 21 condyles, while 7 condyles appeared normal throughout the study period (Table 1). Six condyles demonstrated MRI-defined JOCD lesions at least one timepoint during the study. Of these, three lesions resolved during follow-up (Fig. 3)**.** Two lesions evolved from OV and remained stable throughout the study period without developing symptoms with affected siblings remained under clinical surveillance. One lesion progressed from an OV to an MRI-defined JOCD, subsequently became clinically symptomatic, and ultimately required surgical treatment (Fig. 4).

At the participant level, 6 of 7 siblings exhibited an OV lesion at least once during the study, while 4 of 7 siblings demonstrated MRI-defined JOCD lesions, and 1 of 7 sibling was diagnosed with clinically symptomatic JOCD. At the knee level, OV lesions were observed in 12 of 14 knees at least once, whereas JOCD lesions occurred in 6 of 14 knees over the study period.

### Lesion characteristics

**Fig. 5:**
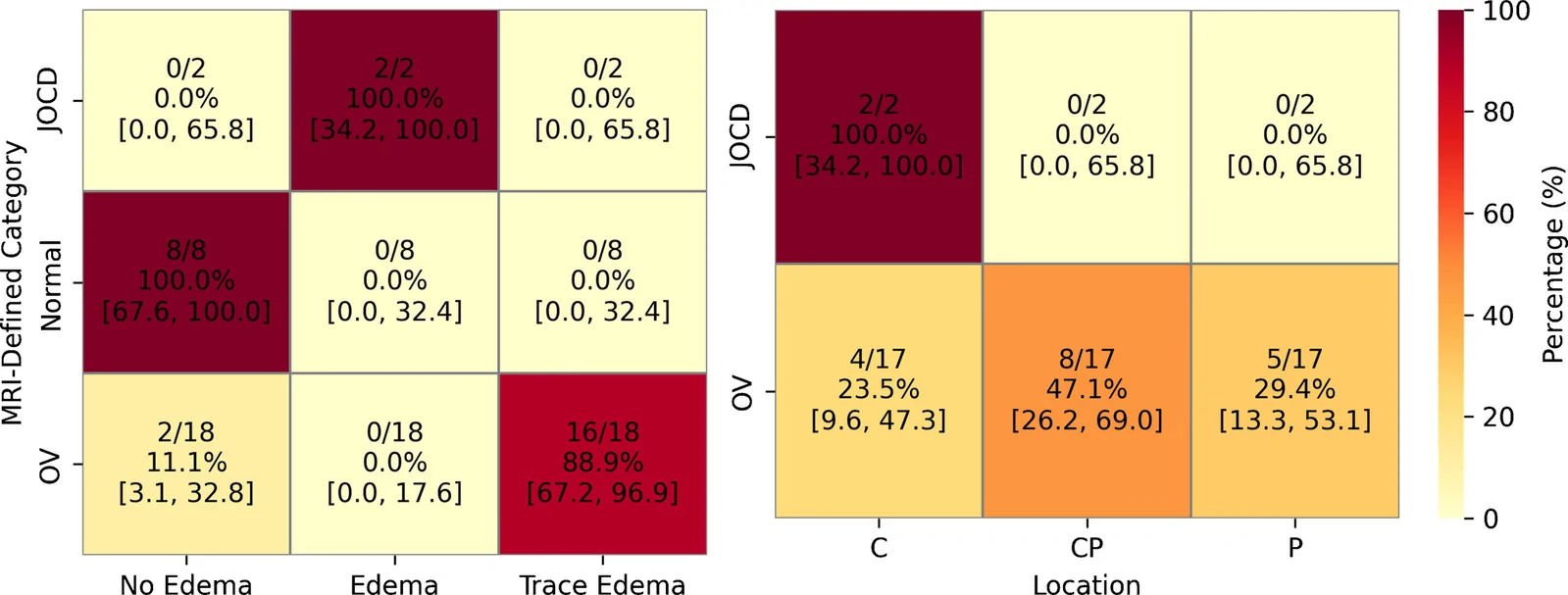
Crosstabulation of MRI-defined category (normal = no lesions, OV lesions, JOCD lesion) versus bone edema (no edema, trace edema, edema) and location (C, CP, P) across all MRI studies including only baseline data. OV = ossification variant, JOCD = juvenile osteochondritis dissecans, C = central, CP = central-posterior, P = posterior

Analysis of condyles across all timepoints showed that bone-marrow-edema characteristics demonstrated distinct distributions across diagnostic categories (Fig. 5). Normal condyles showed no edema in 100% (95% CI: 67–100%) of observations. OV lesions were most frequently associated with trace edema (88%, 95% CI: 67–96%), with fewer observations showing no edema (11%, 95% CI: 3–32%). JOCD was associated with edema (100%, 95% CI: 34.2–100.0%).

OV lesions occurred predominantly in central posterior (47%, 95% CI: 26–69%), and posterior (29%, 95% CI: 13–53%) regions, as traditionally described, but were also seen in central region (23%, 95% CI: 9–47%). JOCD lesions were located in central regions (100%, 95% CI: 34–100%) (Fig. 5).

### Association of the imaging criteria

Analysis of imaging features demonstrated a strong and statistically significant association between bone-marrow-edema and MRI-defined category (OV, JOCD) (F = 31.73, p < 0.001).

**Fig. 6:**
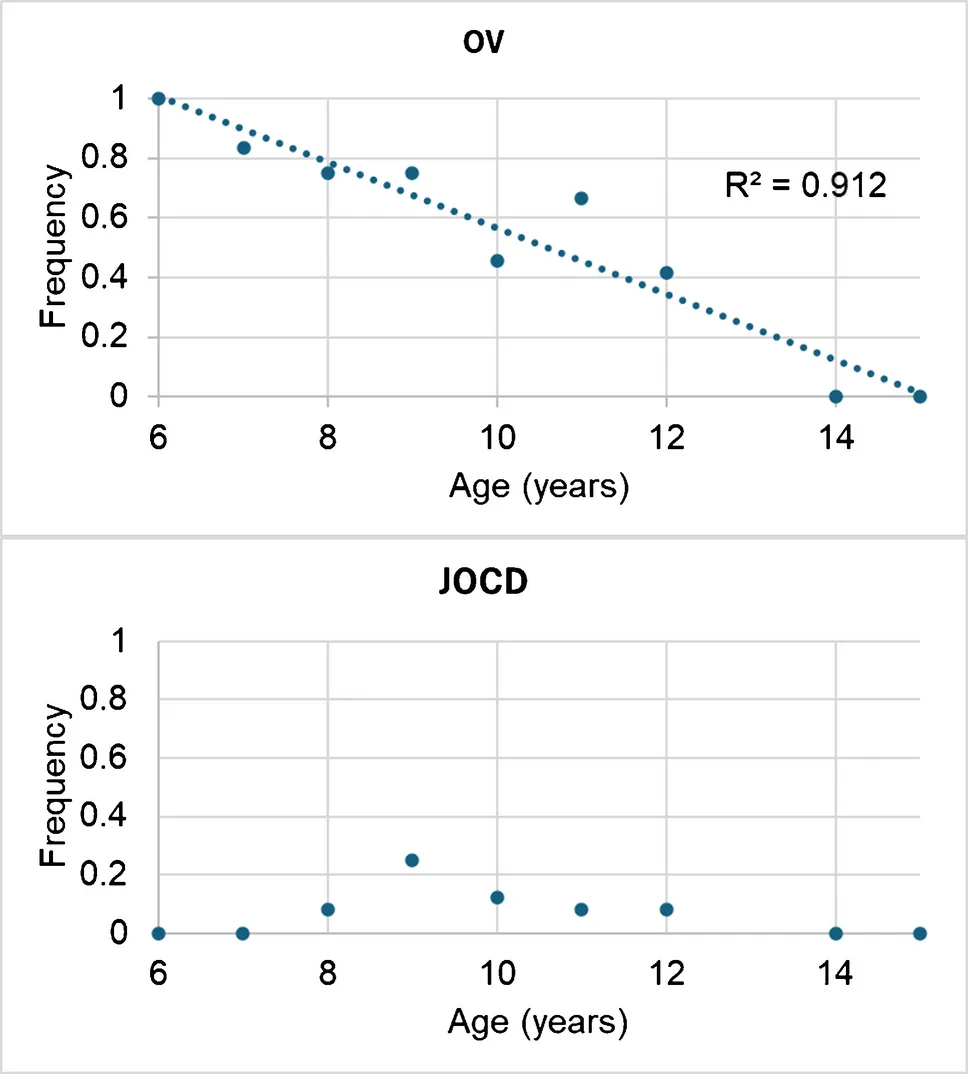
Relationship between age and lesion frequency for OV and JOCD. Data include both baseline and follow-up timepoints from seven siblings. For each age, the total number of OV and JOCD lesions across all subjects and timepoints was counted and normalized to the maximum observed count, and expressed as frequency on the y-axis. OV = ossification variant, JOCD = juvenile osteochondritis dissecans

Analyzing the relationship between the prevalence of OV lesions across condyles at each imaging timepoint and age showed that OV lesions decreased with increasing age, as previously described [3, 17], whereas MRI-defined JOCD lesions were uncorrelated with age (Fig. 6).

### Interrater reliability

The inter-rater agreement between the two musculoskeletal radiologists was substantial for bone edema grading (κ = 0.739, 95% CI: 0.633–0.845, p < 0.001) and MRI-defined category (κ = 0.717, 95% CI: 0.605–0.829, p < 0.001), and moderate agreement for lesion location (κ = 0.479, 95% CI: 0.314–0.644, p < 0.001).

## Discussion

The most important finding of this longitudinal MRI study is that ossification variants (OVs) of the femoral condyles in siblings of juvenile osteochondritis dissecans (JOCD) patients behave dynamically over time and, in rare cases, can evolve into MRI-defined JOCD and even clinically manifest disease. Traditionally, OVs of the femoral condyles and JOCD have been regarded as entirely distinct entities [5]. OV lesions are widely accepted as benign, developmental variants that occur during skeletal maturation and resolve spontaneously without clinical consequence [2–4, 6, 17]. In contrast, JOCD is a pathological process of failed endochondral ossification that may lead to pain, instability, and sometimes require surgery [12–14]. Diagnostic separation has relied on MRI criteria – lack of bone marrow edema beyond red marrow, frequently more posterior location, and greater residual physeal cartilage reflecting younger age for OV lesions versus definite bone marrow edema beyond red marrow adjacent to a delay of the ossification front for JOCD lesions [5, 6].

Our longitudinal 3 T MRI study in asymptomatic siblings of JOCD patients suggests that a strict binary classification of OV versus JOCD lesions on MRI may not always apply in this familially predisposed cohort. OV lesions were highly prevalent in our cohort corresponding to 75% of condyles showing OV or JOCD findings at one or more timepoints. In comparison, unselected MRI pediatric cohorts report prevalences of 12.6–22% [1, 2], suggesting a higher prevalence in this familially enriched cohort, though the small sample size precludes precise estimation. In our study, approximately 6 of 14 knees demonstrated imaging findings of JOCD, including bone marrow edema and delay or irregularity of the ossification front, lesions evolved over time: two progressed to MRI-defined JOCD but remained clinically silent, and one progressed from an OV lesion at baseline to MRI-defined JOCD with onset of symptoms and subsequent development of clinically manifest JOCD requiring surgery. On a participant level, the proportion of siblings with MRI-defined JOCD in our cohort (57%, 4/7) is substantially higher than the 14% family history prevalence reported by Gornitzky et al.[22], but this reflects fundamentally different study designs and diagnostic thresholds rather than contradictory findings. Gornitzky et al.[22] identified clinically symptomatic patients through diagnosis codes and surgical logs, capturing only lesions that had manifested clinically, whereas our protocol detected subclinical lesions invisible to clinical assessment or plain radiographs. Of note, the 1 of 7 siblings (14.3%) in our cohort developed clinically manifest JOCD requiring surgical treatment, consistent with the 14% reported by Gornitzky et al.; the two studies may be complementary rather than contradictory. These findings suggest that, in children with familial association, OVs are not always innocuous but may behave as an early, potentially reversible phase of the JOCD disease process. This interpretation is consistent with recent population-based MRI data, which reported a markedly higher JOCD prevalence of 0.7% compared with 0.095% in earlier radiograph-based cohorts, supporting the notion that JOCD—particularly in its early phase, often minimally symptomatic stage—has been historically underdiagnosed [1].

We are not aware of prior longitudinal pediatric MRI studies documenting progression from an ossification variant to MRI-defined and clinically manifest JOCD in asymptomatic siblings of affected patients. This observation is not consistent with the conclusion of Jans et al. that OVs of the femoral condyles are not associated with osteochondritis dissecans [2], though given the small and non-systematic nature of our cohort this discrepancy should be interpreted with caution. However, our results are consistent with prior histologic work questioning a strict dichotomy between ossification variants and osteochondrosis lesions [23–25] and with Laor’s caution that the long-term relationship between OVs and JOCD remains uncertain [5]. While most OVs in our cohort either remained stable or resolved on MRI, the spectrum of outcomes indicates that not all OV lesions are self-limited. Variable outcomes in children with familial association indicate that OV lesions may represent early stages of the JOCD spectrum, which would warrant follow-up studies. In keeping with prior studies, we observed that OV prevalence in our cohort had a negative correlation with age [3, 17], whereas MRI-defined JOCD lesions showed no apparent age correlation within our cohort, likely reflecting the restricted age window examined (6–10 years at baseline), which predates the peak adolescent incidence of clinically manifest JOCD reported in the broader literature [18, 26].

The crosstabulation analyses confirmed that bone marrow edema was the strongest imaging discriminator between normal condyles, OV, and JOCD lesions. Normal condyles showed no edema in 100% of observations, OV lesions were associated almost exclusively with trace edema and JOCD lesions almost always demonstrated definite edema. As expected, JOCD lesions occurred predominantly in the central region. OV lesions were predominately located central-posterior and posterior as traditionally described. Importantly, both readers demonstrated the same edema–MRI-defined category and location– MRI-defined category relationships, indicating that these patterns reflect lesion biology rather than reader behavior and reinforce the concept of a continuous OV–JOCD spectrum rather than a strict dichotomy.

Longitudinally many lesions did not follow a simple linear path. Lesions initially scored as OV often transitioned between OV, JOCD, and normal imaging findings, and lesions normalized by the end of follow-up. Only a single lesion progressed from imaging findings of OV to MRI-defined JOCD lesion and ultimately to painful clinically manifest JOCD, whereas the other evolving OV lesions remained clinically silent. Importantly, OV lesions that demonstrated evolving marrow edema or increasing delay or irregularity of the ossification front were the lesions most likely to transition toward MRI-defined JOCD, suggesting imaging features that may justify closer surveillance in selected cases.

Even with predefined criteria and two experienced musculoskeletal radiologists, the exact boundary between OV and early JOCD lesions remained challenging. Inter-reader reliability for edema, lesion location, and MRI-defined category was substantial overall, and there were no cases in which one reader labeled findings as normal and the other as JOCD. Disagreements occurred almost exclusively at the OV–JOCD lesion interface: one reader was more “JOCD-sensitive,” assigning JOCD MRI diagnoses earlier and more often, whereas the other was more conservative and retained the label OV for the same lesions. This pattern reflects the inherently subjective threshold contained in the phrase “bone marrow signal beyond red marrow.” Distinguishing trace hyperintensity on fat saturated PD- or T2-weighted TSE images from definite edema in the developing skeleton is influenced by age, marrow composition, sequence parameters, and reader bias, and is precisely where over- and under-calling JOCD lesions – and consequently over- or under-treatment may occur.

Despite these differences in labelling, both readers consistently identified the same knees and lesions at risk; the same left and/or right knees were repeatedly flagged as abnormal in the same siblings, and both readers recognized the single lesion that progressed to symptomatic clinical JOCD. Thus, although nominal MRI-defined categories (OV vs JOCD) varied at intermediate timepoints, the knees that ultimately developed JOCD lesion imaging features were robustly flagged. This convergence suggests that, in children with familial association, an OV accompanied by evolving marrow edema or increasing delay and/or irregularity of the ossification front should not be dismissed as incidental. Instead, our findings support targeted follow-up imaging in selected high-risk patients and knees, acknowledging both the dynamic OV–JOCD spectrum and the limits of single-timepoint MRI.

Patient activity level and sports participation were not collected as part of the IRB-approved study protocol for this asymptomatic research cohort and therefore could not be evaluated. Nevertheless, given the role of mechanical loading in JOCD pathogenesis [12], activity-related factors may plausibly influence progression along the OV–JOCD spectrum. Furthermore, the familial clustering of JOCD observed in our cohort may reflect shared environmental exposures, including household physical activity culture, rather than or in addition to genetic predisposition. These questions warrant evaluation in future and prospectively designed studies.

Our results also align with emerging evidence for genetic susceptibility to JOCD [8, 12, 22, 27]. Mutations in genes such as ACAN, along with familial clustering of JOCD lesions, support a heritable contribution to both the occurrence and behavior of epiphyseal abnormalities [8, 27]. It is plausible that the same familial association that predisposes to JOCD also modulates whether an OV remains a benign developmental variant or progresses along the spectrum toward pathology and may warrant targeted follow-up imaging. Healthcare providers caring for patients with JOCD should be aware of these preliminary findings so that families can be appropriately counseled about the possible increased risk in siblings, particularly given the familial clustering observed in this cohort. While MRI screening of asymptomatic siblings is not currently clinically indicated and is not recommended based on this pilot study alone, awareness of this risk may inform clinical conversations and lower the threshold for investigation when siblings develop knee symptoms.

This study has limitations***.*** The cohort included only seven siblings from a single center and represents a highly selected group with familial risk; therefore, the frequency and behavior of OV lesions observed here cannot directly be generalized to unselected pediatric population. Analyses were lesion-based and performed at the condyle and timepoint level; however, repeated observations within the same participant, though accounted for using generalized linear mixed model, are not statistically independent, and reported associations should be interpreted as descriptive and hypothesis-generating.

Although most JOCD lesions on MRI did not result in clinically manifest disease, the documented progression of one lesion–and transient JOCD imaging findings in several others–demonstrates that progression can occur in children. Future studies should validate these findings in larger, more diverse cohorts with matched controls, incorporate genetic and biomechanical data, and further refine imaging criteria; particularly for marrow edema, to better identify which OV lesions warrant closer surveillance. Several important clinical questions emerge from these observations that warrant investigation in larger, prospectively designed studies. First, whether and under what circumstances clinical examination, plain radiography, or MRI evaluation of siblings of JOCD patients is justified, remains to be determined; the answer will likely depend on symptom status, activity level, and the results of larger cohort studies. Second, the specific combination of genetic predisposition, skeletal maturation stage, and mechanical loading that tips an ossification variant from a benign developmental course toward clinically manifest JOCD remains unknown. Identifying these modifiers is essential for distinguishing siblings who require active surveillance from those who can be reassured.

In conclusion, our preliminary findings in this small pilot cohort, conducted exclusively within an IRB approved research setting and not intended as a recommendation for clinical screening, suggest that in children with familial association, OV lesions may represent early stages along a continuum of JOCD lesions rather than a separate benign entity. These observations require validation in larger, prospectively designed studies. If clinically indicated, greater radiologist awareness and consistent application of MRI criteria, particularly with respect to marrow edema, may refine diagnosis and enable risk-stratified follow-up and timely orthopedic evaluation, facilitating earlier, often nonoperative intervention when JOCD lesions on imaging show progression to clinically manifest disease. Prospective studies including larger sibling cohorts are needed to determine whether risk-stratified counseling and structured follow-up can reduce the burden of advanced JOCD in this familially predisposed population.

## Data Availability

Not applicable.
